# Hemophagocytic lymphohistiocytosis during treatment of intracranial multifocal germinoma: a case report and literature review

**DOI:** 10.3389/fonc.2024.1264926

**Published:** 2024-03-12

**Authors:** Ting Guo, Zichun Liu, Yixin Chen, Yangyang Cheng, Kaitong He, Xin Lin, Mingzhu Wang, Yihua Sun

**Affiliations:** ^1^ Department of Clinical Laboratory, Fengcheng Hospital of Fengxian District, Shanghai, China; ^2^ Department of Clinical Laboratory, Harbin Medical University Cancer Hospital, Harbin, China

**Keywords:** hemophagocytic lymphohistiocytosis, hemophagocytic syndrome, malignancy, intracranial multifocal germinoma, case report

## Abstract

Hemophagocytic lymphohistiocytosis (HLH), also known as hemophagocytic syndrome (HPS), is a benign histiocytosis with hyperreactive proliferation of the mononuclear phagocyte system caused by immune function abnormalities, which often occurs under the background of genetic mutations, inflammation, infection or tumors. Because the research on malignancy-associated HLH (M-HLH) is focused on hematological malignancies, reports on HLH secondary to solid tumors are rare. In this case, we report a 14-year-old girl who developed HLH during treatment for intracranial multifocal germinoma, and the disease was controlled after hormone combined with etoposide(VP-16) and other related treatments. To our knowledge, there have been no documented cases of HLH caused by intracranial multifocal germinoma.

## Introduction

Hemophagocytic lymphohistiocytosis (HLH) is a severe and even life-threatening syndrome of excessive inflammatory response due to impaired activity of cytotoxic T lymphocytes and natural killer cells (1). It is characterized by unregulated activation and proliferation of macrophages in all reticuloendothelial organs (e.g., bone marrow, spleen, liver, and lymph nodes), leading to a persistent cytokine storm and histiocytosis, with clinical symptoms such as persistent fever, pancytopenia, and hepatosplenomegaly, and rapid progression to disseminated intravascular coagulation and multiple organ failure (2). HLH can be divided into Primary HLH(P-HLH) and Secondary HLH(S-HLH). P-HLH mainly occurs in children aged 0-2 years with poor prognosis,which is autosomal or sex chromosome recessive genetic disease (2). However, S-HLH usually occurs in adults without genetic factors, which often induced by infection, malignancy, hematopoietic stem cell transplantation, autoimmune diseases, immunodeficiency diseases, and drug hypersensitivity reactions. The number of reports on Malignancy associated hemophagocytic syndrome(M-HLH) has been increasing year by year. However, the current known studies of M-HLH are still focused on hematological malignancies, such as lymphoma, acute leukemia, myelodysplastic syndrome, etc., while S-HLH in malignant solid tumors is rarely mentioned.

Here, we give a report of a secondary HLH case in a 14-year-old girl during her treatment of intracranial multifocal germinoma. The patient developed severe intracranial infection after craniotomy. During her chemoradiotherapy, there were pancytopenia, transient liver injury, persistent fever, exfoliative dermatitis, and bone marrow hemophagocytosis, etc. But the final prognosis was good after early diagnosis and early therapy. According to the onset time of HLH in this case, it is suggested that tumor cell destruction induced by chemoradiotherapy drugs and secondary infection after immunosuppression may be the trigger factors of HLH. At the same time, we review the relevant literatures on malignant tumor-associated hemophagocytic syndrome and give the report of this case.

## Case description

**Figure 1:**
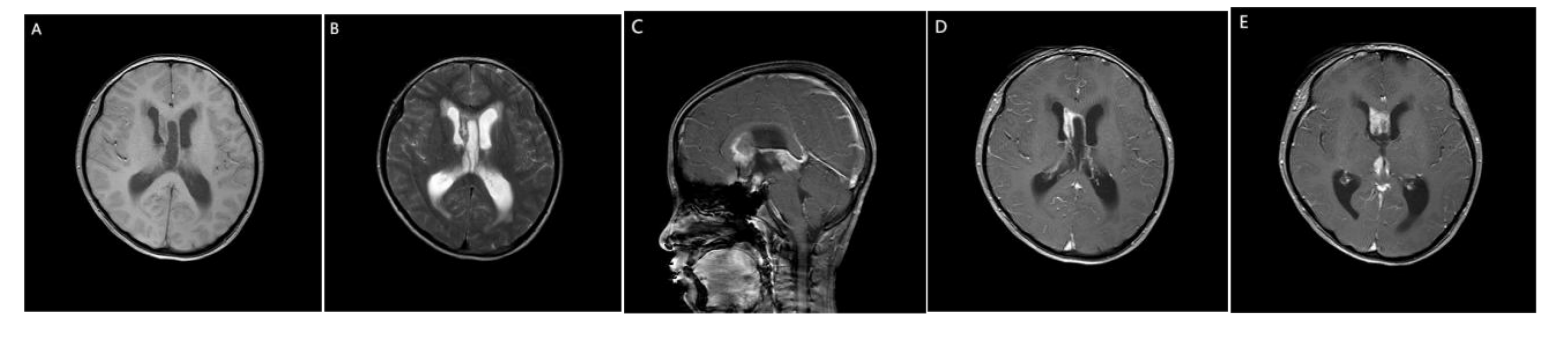
MRI of the patient’s brain. **(A)** Slightly hypo signal on T1WI. **(B)** Slightly higher signal intensity on T2WI. **(C)** sagittal view. **(D, E)** On enhanced scan, the nodules were significantly enhanced, and the lesions grew along the ventricular wall casting.

**Figure 2:**
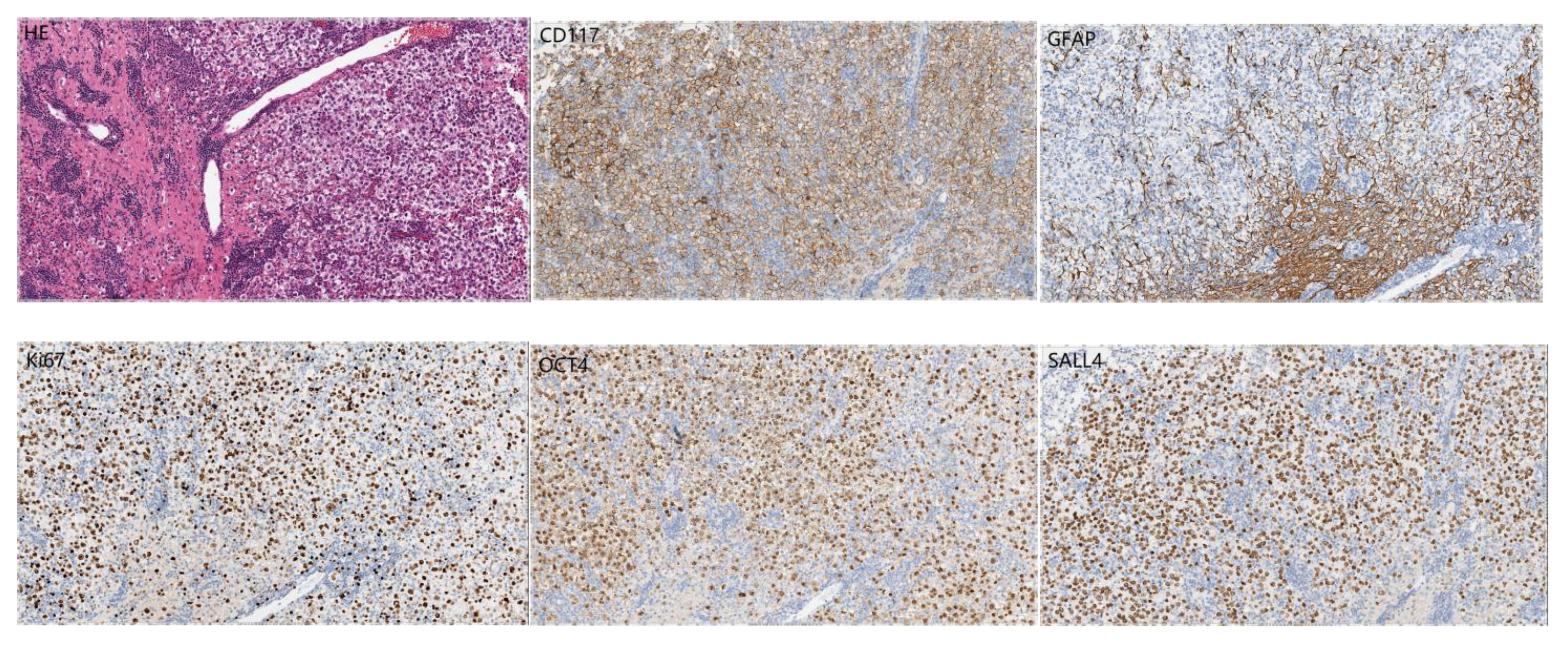
Postoperative pathological images of the tumor ×200.

A 14-year-old girl came to Harbin Medical University Cancer Hospital due to intermittent headache for 10 days without obvious incentive on December 28, 2020. On admission, she denied special life history, family history of previous diseases or genetic diseases and any accompany symptoms of nausea, vomiting, dizziness. She had stable vital signs, dysplasia, and not yet menarche. The height of the patient was 150cm, the weight was 29kg, and the BMI was only 12.89kg/m^2^. The rest of the patient’s medical and neurological examinations showed no abnormalities. Imaging examination (December 24, 2020): Head enhanced Magnetic Resonance Imaging (MRI) (**Figure 1**) showed irregular nodular shadows in the intracranial pineal region, septum pellucidum, lateral ventricle and suprasellar cistern, the larger one was about 16 ×16 mm in size, and the lesion grew cast along the ventricular wall; the bilateral ventricle and third ventricle revealed hydrops; the septum pellucidum showed dilatation. Due to the multiple periventricular masses in this patient, ependymoma was considered as a possibility, and germ cell tumor could not be ruled out. Electrocardiogram, chest CT and echocardiography displayed no abnormalities. Serological tests: sodium 153 mmol/L (reference range:137 ~ 147 mmol/L), chlorine 112 mmol/L (99 ~ 110 mmol/L), high-density lipoprotein cholesterol1.83 mmol/L (1.04 ~ 1.74 mmol/L), homocysteine 27.5 ummol/L (1.5 ~ 15 ummol/L), prolactin 97.73 ng/ml (female: 5.18-29.53 ng/ml), alpha-fetoprotein 2.16 ng/ml (0-7 ng/ml), carcinoembryonic antigen 1.29 ng/ml (0-5 ng/ml), human chorionic gonadotropin β subunit 0.69 mIU/ml (0-5 mIU/ml); Other laboratory parameters [three infectious disease antibodies (human immunodeficiency virus, hepatitis C virus, treponema pallidum), five hepatitis B, and coagulation function] were in the normal range. Urine routine: urine specific gravity 1.003 (1.015-1.025). Cerebrospinal fluid(CSF) examination: lateral decubitus pressure 180 mmH_2_O, colorless and transparent, protein (+), white blood cell count 10 × 10^6^/L, monocyte percentage 4.9%, lymphocytes 90.2%, AFP < 0.61 ng/ml (reference range:0-7 ng/ml), β-HCG 15 mIU/ml (0-5 mIU/ml), and no atypical cells on cytology. On January 4, 2021, the patient underwent craniotomy with tumor resection near the intercompartment hole area of the septum pellucidum, posterior part of the third ventricle, and quadrigeminal region, as well as third subventricular fistula. Postoperative pathological immunohistochemical markers (**Figure 2**): SALL4 (+), CD117 (+), OCT4 (+), D2-40 (+), CD30, GFAP, S-100, Syn, LCA, AFP and CYP-3 were all negative, and Ki67 labeling index was about 80%, which was diagnosed as germinoma. MRI of the whole spine showed that the physiological curvature of the patient’s cervical spine was straightened, and there was no abnormality in the rest. After surgery, the patient was given etoposide combined with carboplatin regimen chemotherapy for 1 cycle, during which she developed bone marrow suppression, severe intracranial infection and transient liver injury (ALT: 166U/L, AST: 422U/L, LDH: 4575U/L). After a series of symptomatic treatments, the patient ‘s condition was improved and underwent sequential IMRT radiotherapy in February 2021. Combined with image-guided techniques, irradiation was performed for the whole brain spinal cord and tumor bed to complete the whole spinal cord DT: 1600 cGy/10f, whole brain DT: 3600 cGy/20f, and tumor bed area boost DT: 1400 cGy/7f. During radiotherapy, the patient developed grade IV bone marrow suppression, which had reached the indication of blood transfusion, and the patient’s hemogram recovered after multiple treatments of leukocyte-elevating platelets and blood transfusion. Then the patient could not tolerate whole brain and spinal cord radiotherapy, so the plan was changed to continue radiotherapy for the whole brain and tumor bed area.

**Figure 3:**
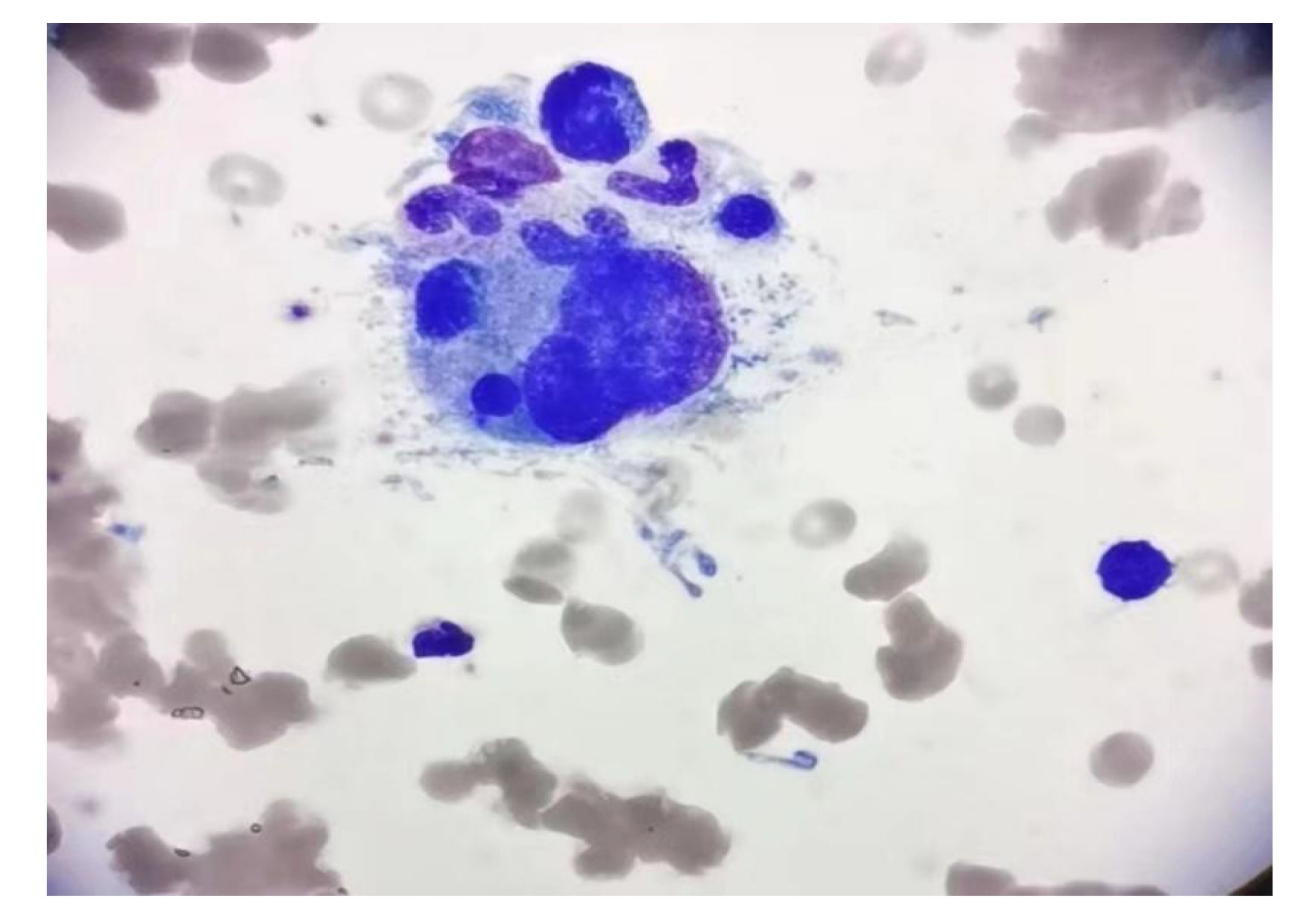
Bone marrow aspirate smear showed the evidence of hemophagocytosis; original magnification ×1000.

The patient was readmitted on 21 May 2021 with pancytopenia. Laboratory tests: pancytopenia (white blood cell (WBC)2.12 × 10^9^/L, red blood cell count(RBC) 2.69 × 10^12^/L, hemoglobin (HB)89 g/L, platelet count(PLT) 20 × 10^9^/L); ion disturbance (potassium 3.1 mmol/L, sodium 184 mmol/L, chlorine 144 mmol/L, magnesium 1.26 mmol/L); mild liver dysfunction (lactate dehydrogenase 322 U/L), decreased serum total protein 63.7 g/L, urine routine white blood cell 20.9/ul, normal coagulation, and no tumor metastasis or recurrence on brain MRI. The patient’s peripheral blood was extremely reduced, which was considered to be related to bone marrow suppression after chemoradiotherapy, and bone marrow invasion was not excluded. The patient was given WBC-elevating needle, transfusion of leukocyte-depleted platelets and suspended red blood cells. On May 25, the platelet increased to 91 × 10^9^/L and bone marrow aspiration was performed immediately to determine whether there was bone marrow infiltration. Bone marrow aspiration (**Figure 3**): grade III myelodysplasia, a few cells of multiple lineage abnormal development, easy to see hemophagocytic cells. We highly suspected that the patient had secondary hemophagocytic syndrome and immediately performed relevant tests: ferritin 351.8 ng/ml on May 27. According to experience, we immediately gave the patient intravenous dexamethasone (10 mg/d × 13 days) combined with etoposide (50 mg/d × 3 days) regimen, and transfused red blood cells and leukocyte-depleted platelets, subcutaneous thrombopoietin and granulocyte colony-stimulating factor and other supportive treatment, closely monitored the hemogram and timely adjusted the treatment regimen. On June 2, the patient had persistent fever up to 38.4deg;C, perfected relevant infection tests (G test, blood culture, sputum and urine bacterial culture were negative, procalcitonin 0.681 ng/ml), and was given intravenous meropenem (1.5 g/d × 14 d) and fluconazole (100 ml/d × 13 d) for anti-infective treatment. On June 17, blood routine (WBC 7.96 × 10^9^/L, RBC 2.68 × 10^12^/L, Hb 85 g/L, Plt 86 × 10^9^/L) and ions (potassium 3.4 mmol/L, sodium 166 mmol/L, chloride 127 mmol/L) were reexamined, and the patient strongly requested to be discharged. Within the following 6 months, the patient was admitted several times due to pancytopenia, and hemophilic cells was still observed in the bone marrow three times successively. Fortunately, intravenous VP16 (50 mg/d × 3 days) combined with steroids (10 mg/d × 3 days) kept the patient ‘s condition under good control. During this time, the patient presented with subcutaneous bleeding, coagulation abnormalities (fibrinogen 4.88g/L, elevated D-dimer 1.11mg/L), keratolysis exfoliative of both hands, and persistent hypersodium, hyperchloride, and hypokalemia. At the time of discharge from hospital on January 23, 2022 to the last follow-up on August 30, 2022, the patient was in good condition and had not received any further treatment.

## Discussion

Intracranial germ cell tumors (GCTs) are rare malignant brain tumors derived from primitive embryonic cell, accounting for 1% to 2% of primary intracranial tumors in North America and Europe, while Asians have a higher incidence compared with Westerners (3). GCTs is more common in children and young adults, with a male to female ratio of approximately 2:1, which often appears in the form of solitary nodules or multiple lesions[3]. Different histological types of GCTs often occur on midline structures of the central nervous system, particularly in the pineal gland (48%) and suprasellar (neurohypophysis) region (37%). GCTs away from the midline, such as the basal ganglia, thalamus, cerebellar vermis, ventricular system, and optic chiasm, may be caused by the migration of ectopic germ cell from the midline during ventricular development, but no studies have yet revealed its etiology (3, 4). Germinomas are the most common type of GCTs, with about 10% of GCTs presenting as bifocal tumors (involving both the pineal gland and neurohypophyseal region), and multifocality is even rarer (5). No studies have clearly demonstrated whether this phenomenon is caused by tumor metastasis along the cerebrospinal fluid or synchronous tumor growth. American scholars believed that it is metastatic disease, but it was considered to be a localized disease in Europe (6). When germinoma is disseminated and metastasized in the ventricular system, it usually presents as nodular abnormally enhanced lesions along the ependyma, which should be differentiated from ependymoma (7). In this case, no dysmorphic cells were observed in preoperative cerebrospinal fluid liquid-based smear, and imaging examination could not confirm the diagnosis so surgical treatment was adopted. In addition, as for the pathogenesis of germinoma, some studies have shown from the molecular biological level that primary intracranial multifocal germinoma has multiple chromosome imbalances, and almost all of them have manifested hypomethylation and active X chromosomes, which indicates that the occurrence of Intracranial multifocal germ cell tumors (IMGCTs) is related to chromosomal changes (8). In this case, due to the patient’s young age and multiple lesions, even if no abnormal cells were found in the cerebrospinal fluid, we were unable to completely rule out metastasis. For long-term prognosis considerations, we initially administered craniospinal radiation therapy. However, for the entire spinal cord, we only used low-dose prophylactic radiation (1600cGy/10f). When the patient later found it difficult to tolerate craniospinal radiation and developed IV degree bone marrow suppression, we changed the plan to only continue irradiation for the entire brain and tumor bed.

The various clinical manifestations of germinoma are related to the size, the site, and the invasion extent of the tumor. Tumors located in the pineal region often block the midbrain corpora quadrigemina and cause hydrocephalus due to tumor protruding to the back of the third ventricle or invading the tetras downward, thus leading to headache, vomiting and other symptoms of elevated intracranial pressure, which can also be manifested as binocular hyperopia (Parinaud syndrome) (9). Germinoma in sellar region or suprasellar region is often accompanied by diabetes insipidus, precocious puberty or delay, hypothyroidism, growth hormone deficiency, adrenal insufficiency and other endocrine disorders, which may invade optic chiasma and cause vision loss or visual field defect (10). In this case, the patient was admitted with intermittent headache as the first symptom, without obvious symptoms of polyuria and polydipsia. Examination showed a decreased urine proportion, accompanied by hypernatremia and abnormal neuroendocrine hormones. Oral DDAVP was effective, hence central diabetes insipidus was considered. The tumor invaded the anterior inferior wall of the third ventricle of the patient, damaging the vascular endplate in the anterior hypothalamus, namely the osmotic pressure receptor in the AV3V region and the thirst center. The supraoptic nucleus was damaged and the hypothalamic-pituitary axis was destroyed, resulting in reduced vasopressin secretion and increased PRL secretion. Meanwhile, the repeated existence of low potassium, high Na and high K during the course of the disease is also related to the treatment of cranial pressure reduction by dehydration. In addition, the patient presented with intermittent headache as the first symptom, which may be caused by hydrocephalus or GCT in the septum pellucidum. Besides connecting two cerebral hemispheres, the pellucidum has no other clear function. Studies have shown that tumors here often take the symptoms and signs of headache and epilepsy as the first symptom (11). At present, there are only individual reports on septum pellucidum germ cell tumor, which needs further exploration.

Hemophagocytic lymphohistiocytosis (HLH) is a rare and serious inflammatory disorder characterized by excessive activation of immune cells, resulting in impaired pathogen clearance, sustained activation of the immune system, and massive production of cytokines (interleukin (IL) -1-β, IL-2, IL-6, IL-10, IL-18, and tumor necrosis factor (TNF), etc.) (12). Depending on the trigger, HLH can be classified as primary or hereditary and secondary. Primary or hereditary HLH is characterized by a number of different genetically heterogeneous diseases caused by mutations in high-permeability genes that affect cytolytic function, lymphocyte survival, or inflammasome activation, including familial HLH (pathogenic alterations in PRF1, UNC13D, STXBP2, and STX11 lead to severe impairment of NK and CD8+ cell cytotoxic function), certain dyschromic immunodeficiency diseases (RAB27A, LYST, AP3B1 mutations resulting in degranulation disorders, such as Griscelli syndrome type II and Chediak-Higashi syndrome), X-linked lymphoproliferative disorders (SH2D1A and XIAP gene defects), and other diseases such as NLRC4, CDC42, and eb virus susceptibility (12–14). Secondary HLH does not have any obvious genetic tendency of HLH, and is often induced by infection, malignant tumor, and autoimmune diseases. Interestingly, HLHs in different disease contexts have different terminologies. It is commonly called macrophage activation syndrome (MAS) when it occurs in the context of rheumatic or autoinflammatory diseases, and cytokine release syndrome is commonly caused by bispecific T-cell engager (BiTE) or chimeric antigen receptor (CAR) therapy (14, 15). Infection-associated HLH is common with DNA viruses (Epstein-Barr virus, cytomegalovirus, and adenovirus) and intracellular pathogens (leishmaniasis), and there have been reports of HLH caused by brucellosis and COVID-19 infection, as well as cases of HLH caused by COVID-19 vaccination (16–19). M-HLH is common in hematologic tumors, such as T-cell or NK-cell lymphomas or leukemias, followed by B-cell lymphomas, and HLH caused by solid tumors is rare (20). There have been numerous previous reports on M-HLH, such as gastric cancer (21), glioblastoma (22), prostate cancer (23), and pembrolizumab in the treatment of head and neck squamous cell carcinoma to induce HLH (24). Here we report a case of intracranial germinoma causing HLH.

Malignancy plays a pathogenic role in approximately 50% of adult HLH. HLH may emerge as a consequence of malignancy, occurring before or during cancer therapy and can complicate malignancy in 1% of adults with very high mortality (25). At present, the pathological mechanism of M-HLH is still unclear, and inflammation, as a hallmark of cancer, may play an important role (26). M-HLH is considered to be associated with sustained antigenic stimulation of tumor cells and secretion of large amounts of cytokines, causing excessive inflammatory responses (27, 28). It’s worth noting that chemotherapy-associated HLH(Ch-HLH) occurs well during cancer treatment, including induction, consolidation, and maintenance phases, and is associated with secondary infections caused by immunosuppression caused by chemotherapy (27, 29). Because of the low incidence of M-HLH and the substantial overlap between HLH and tumor characteristics, it makes it challenging to identify HLH in the context of malignancy. The widely adopted pediatric HLH-2004 guideline has strict diagnostic criteria, including positive gene/mutation detection, or in the absence of genetic testing, at least 5 of 8 clinical laboratory tests can confirm the diagnosis (see **Table 1** for details) (30). However, HLH-2004 guidelines have limited diagnosis in adult HLH, and the HLH probability score (H-score, **Table 2**) [http://saintantoine.aphp.fr/score/] can assist in the diagnosis of HLH] (31, 32). Furthermore, the study have shown that HLH-2004 criteria (meeting at least five items) has 91% sensitivity and 93% specificity for predicting HLH, while H-score cut-off value ≥ 169 has 96% sensitivity and 71% specificity for HLH diagnosis (33). Some scholars have also proposed that HLH that cannot be diagnosed but has supportive features including elevated liver transaminases, bilirubin, lactate dehydrogenase (LDH), and D-dimer, while most HLH with seizures as the main clinical manifestation have cerebrospinal fluid leukocytosis and peripheral blood mononucleosis (2). HLH should be highly suspected when patients present with pancytopenia, elevated ferritin, hepatosplenomegaly, and hemophagocytosis in bone marrow or lymph nodes (34). In this patient, the whole blood cells decreased during postoperative chemoradiotherapy for intracranial germinoma, accompanied by persistent fever, elevated ferritin, easy to see hemophagocytosis in bone marrow aspiration and other manifestations, so the occurrence of HLH was rapidly highly suspected. Given the rapid progression of HLH, we can be sure that the patient had HLH even if the test results at that time met only 3 of the 8 diagnostic criteria of HLH-2004 (see **Table 1**). The effectiveness of the subsequent VP-16 combination steroid regimen for HLH and the presence of hemophagocytes in three bone marrow aspirates for three consecutive months also confirmed the accuracy of this diagnosis. Unfortunately, the detailed examination of HLH was not perfect due to the patient ‘s family economy, and serum ferritin, plasma fibrinogen, and fasting triglycerides, which were slightly changed at the initial stage of HLH, were not continuously monitored subsequently. During chemoradiotherapy, the patient developed recurrent pancytopenia, while bone marrow aspiration showed no tumor infiltration, which reflected the destruction of tumor cells by chemoradiotherapy and the inhibition of bone marrow hematopoiesis. It is worth thinking that the tumor burden is relatively small due to tumor resection, and based on the time of onset of HLH, we highly suspect that HLH is caused by secondary infection due to chemoradiotherapy-induced IV bone marrow hematopoietic suppression and immunosuppression. However, the influence of surgical stress and the modulation of cytokines by the primary lesion cannot be completely ruled out. Although infection caused by immunosuppression was considered to cause HLH, the relevant laboratory tests failed to find microbiological evidence. The patient’s condition improved after initial treatment with meropenem combined with fluconazole to cover gram-negative and fungal conditions, suggesting that the infected microorganism was not common, but direct evidence could not be obtained. For GCTs with malignant cell burden, HLH should be considered as a serious adverse event during chemoradiotherapy. Unfortunately, there have been no reports and studies on HLH associated with intracranial germinoma. It has not been confirmed whether it is germinoma itself, or activated immune cells responding to tumor antigens or infection. Even which kind of cell is the main cells that releases cytokines leading to high inflammatory response is a mystery.

**Table 1:** Diagnostic criteria for HLH.

HLH-2004 Diagnostic criteria^[3]^ (Patients need to be greater than or equal to five of the eight diagnostic criteria)	Patient's early symptoms and laboratory findings
Fever (Temperature>38.5°C for>7 day)	*
Splenomegaly /hepatomegaly/ lymphadenopathy	–
Cytopenia (affecting≥2 lineages in peripheral blood)	* (Hemoglobin 89g/l Platelets 16×10^9^/l Neutrophil 1.06×10^9^/l)
Hypertriglyceridemia (>3 mmol/1) and/or hypofibrinogenemia (<1.5g/l)	Triglyceride 1.17 mmol/l Fibrinogen2.55 g/l
Hemophagocytosis (in bone marrow or spleen or liver or lymph node or other tissues)	* (Hemophagocytosis in bone marrow)
Hyperferritinemia (>500μg/l)	351.8 ng/ml
Elevated soluble CD25 (>2400 U/ml)	–
Reduced or absent NK cytotoxicity	–
Supportive evidence	
Elevated transaminases and bilirubin	* (transient)
Elevated lactate dehydrogenase	*
Elevated d-dimers	*
Elevated cerebrospinal fluid cells and/or protein	*

Diagnostic criteria for HLH (-:Untested experimental items for patients, *: The patient met this diagnostic criteria).

**Table 2:** HScore (probability score) in diagnosis of HLH.

HScore (probability score) in diagnosis of HLH^[4]^.	
Judge item	Scoring criteria
Known underlying immunodepression	0 (N) or 18(Y)
Maximal Temperature(°C)	0 (< 38.4), 33 (38.4–39.4), or 49 (> 39.4)
Hepatomegaly/ Splenomegaly	0 (N), 23 (either), or 38 (both)
Reduction level of Hemoglobin/ Leucocytes/ Platelets count	0 (1 lineage), 24 (2 lineages), or 34 (3 lineages)
Higher Ferritin level (ng/ml)	0 (< 2000), 35 (2000–6000), or 50 (> 6000)
Higher Triglyceride level (mmol/l)	0 (< 1.5), 44 (1.5–4), or 64 (> 4)
Lower Fibrinogen level (g/l)	0 (>2.5), or 30 (≤2.5)
Higher serum glutamate oxaloacetate transferase (SGOT) level (UI/L)	0 (<30), or 19 (≥30)
Hemophagocytosis features on bone marrow aspirate	0 (N) or 35 (Y)

N, (No); Y, (Yes).

HLH has diverse clinical manifestations and high mortality, and early diagnosis and treatment are essential. Current treatment goals for HLH are to measure disease severity while taking prompt control of inflammation and addressing any identified triggers based on the underlying etiology or trigger (35). P-HLH is usually treated with steroids and chemotherapy to suppress the systemic immune system, and cure can only be achieved by allogeneic hematopoietic stem cell transplantation (HSCT) (36). MAS was treated with high-dose intravenous corticosteroids (CS) and targeted IL-1 blockade. It is inconclusive whether M-HLH therapy is mainly to inhibit cytokine storm or to treat tumors, or both, and specific analysis is required according to the patient ‘s clinical condition. In the active stage of HLH, especially in patients with organ dysfunction, standard chemotherapy regimens for malignant tumors do not improve disease status at this time, but may increase mortality (37). For patients with M-HLH, dexamethasone/etoposide based HLH-94 regimen/HLH-2004 or DEP regimen is recommended before tumor-specific treatment (30, 38). If the central nervous system is involved, dexamethasone is preferred to better cross the blood-brain barrier, or in combination with methotrexate (39). In this case, dexamethasone combined with etoposide (VP16) was used to control HLH, which was effective and the patient’s condition was significantly controlled. Currently, there is no recognized salvage therapy for refractory HLH, in addition to HLH-94 regimen, and currently effective tentative treatment regimens include the combined use of JAK1/2 inhibitor lusoritinib (40), monoclonal anti-CD52 antibody alemtuzumab (41), neutralizing antibody against INFγ epavatinib (42), and plasma exchange (43). However, the exact location of these regimens in the treatment of HLH remains to be determined.

In summary, we report a case of HLH following surgical, chemoradiotherapy for intracranial multifocal germinoma. Early diagnosis and treatment of HLH significantly inhibited the dramatic deterioration of the disease. In the future, further studies are needed to clarify the pathogenesis of HLH in cancer patients. Simultaneously, it is imperative to find appropriate treatment options for refractory HLH.

## Data availability statement

The datasets presented in this article are not readily available because requests to access the datasets should be directed to Ting Guo,guot202206@163.com.

## Ethics statement

The studies involving humans were approved by the ethics committee of the Harbin Medical University Cancer Hospital (Harbin, China). The studies were conducted in accordance with the local legislation and institutional requirements. Written informed consent for participation was not required from the participants or the participants’ legal guardians/next of kin in accordance with the national legislation and institutional requirements. Written informed consent was obtained from the individual(s), and minor(s)’ legal guardian/next of kin, for the publication of any potentially identifiable images or data included in this article.

## Author contributions

SYH designed the study. GT gathered the clinical information and drafted the manuscript. LZC, CYX, CYY, and HKT confirm the authenticity of all raw data. LX and WMZ revised the manuscript. All authors contributed to the article and approved the submitted version.
